# Acetaminophen-induced hypotension in sepsis

**DOI:** 10.1186/s40780-022-00245-y

**Published:** 2022-05-03

**Authors:** Shunsuke Inage, Ryo Yajima, Shintaro Nagahara, Aya Kazama, Moe Takamura, Tomohiro Shoji, Mika Kadoi, Yukiko Tashiro, Yuya Ise

**Affiliations:** Department of Pharmaceutical Services, Nippon Medical School Hospital, 1-1-5 Sendagi, Bunkyo-ku, Tokyo, 1138603 Japan

**Keywords:** Acetaminophen, Mannitol, Blood pressure, Hemodynamics, Sepsis

## Abstract

**Background:**

Acetaminophen-induced hypotension has been reported in critically ill patients; however, it remains unclear whether mannitol, present as a stabilizing compound in acetaminophen formulations, affects hemodynamic changes. The objectives of this study were to clarify the direct effect of acetaminophen on blood pressure by comparing blood pressure changes after acetaminophen and intravenous immunoglobulin (IVIG) administration, both containing mannitol, in patients with sepsis and understand the risk factors for reduced blood pressure following acetaminophen administration.

**Methods:**

This was a retrospective cohort study. Adult patients who were diagnosed with sepsis at Nippon Medical School Hospital, and who were undergoing continuous arterial blood pressure measurement and received intravenous acetaminophen or IVIG, were included.

**Results:**

Overall, 185 patients were included, with 92 patients in the IVIG group and 93 in the acetaminophen group. The incidence of hypotension was 36.9% in the IVIG group (34 of 92 patients) and 58.0% in the acetaminophen group (54 of 93 patients) (OR = 8.26, *p* = 0.004). In a propensity score-matched cohort, 80 matched patients were selected. The incidence of hypotension was 37.5% in the IVIG group (15 of 40 patients) and 67.5% in the acetaminophen group (27 of 40 patients) (OR = 7.21, *p* = 0.007).

**Conclusions:**

Acetaminophen induced substantially greater hypotension than IVIG in patients with sepsis, with both containing mannitol. Further studies are needed to clarify the effects on hemodynamics of mannitol contained in acetaminophen formulations.

## Background

Intravenous acetaminophen (also known as paracetamol) is the most widely used antipyretic and analgesic drug during intensive care worldwide [1]. Recently, in critically ill patients, including those presenting severe infections, acetaminophen administration has been associated with a decrease in blood pressure [2–10] and an increased incidence of hypotension lasting for approximately 2 h [4, 5]; however, investigations are small and results are variable. Among critically ill patients with sepsis, those who develop hypotension have an increased risk of mortality [11]. Furthermore, an intravenous acetaminophen formulation (Acelio Intravenous Injection; Terumo Co. Ltd., Tokyo, Japan) contains 3.85 g (0.0385 g/mL) of mannitol, as a stabilizing compound, per 1000 mg of acetaminophen [12]. It has been postulated that the diuretic effect of mannitol could induce a decrease in blood pressure in critically ill patients following the administration of intravenous acetaminophen [2, 13]; however, the impact of mannitol present in acetaminophen formulations on blood pressure remains unclear.

Intravenous immunoglobulin (IVIG) is widely used for the treatment of severe infections during intensive care; approximately two-thirds of physicians in Japan use IVIG as an adjunctive treatment of sepsis [14]. Polyethylene glycol-treated human immunoglobulin formulations for intravenous injection (Kenketu Glovenin-I; Nihon Pharmaceutical Co. Ltd., Tokyo, Japan) reportedly contain 1.5 g (0.015 g/mL) of mannitol as a stabilizing compound per 5000 mg of polyethylene glycol-treated human immunoglobulin, and are usually administered at 5000 mg per day for 3 days in Japan [15]. IVIG affects hemodynamics via an allergic reaction; however, this occurs rarely, and no direct effect of IVIG on blood pressure has been reported [16]. It is possible to clarify the direct effect of acetaminophen on blood pressure by comparing blood pressure changes observed after the administration of acetaminophen and IVIG, both of which contain mannitol, in patients with sepsis. The objectives of this study were to clarify the direct effect of acetaminophen on blood pressure and understand the risk factors for hypotension induced by acetaminophen administration in an ICU population of patients with sepsis.

## Materials and methods

### Study design and setting

This single-ICU, retrospective cohort study was conducted at Nippon Medical School Hospital, a tertiary academic hospital in Tokyo, Japan. This study was reviewed and approved by the Institutional Review Board of Nippon Medical School Hospital (R1-09–1193). The Institutional Review Board waived the need for informed consent.

### Study population

Adult patients with sepsis (defined according to the Society of Critical Care Medicine Sepsis-3 criteria) [17] hospitalized during the period from September 1, 2014 through August 31, 2019 at Nippon Medical School Hospital, and who were undergoing continuous arterial blood pressure measurement and who received IVIG or intravenous acetaminophen, were included. Arterial blood pressures were automatically and continuously recorded. Patients were excluded for the following reasons: (1) started or increased the use of sedatives during the observation period, (2) started or increased the use of hypotensive agents during the observation period, (3) started or increased the use of analgesic agents during the observation period, (4) discontinued or reduced the use of vasopressors during the observation period, (5) received intravenous mannitol between 24 h before and 2 h after the administration of the target drug, (6) received another target drug (IVIG or acetaminophen) during the observation period, and (7) had missing data. In the case of multiple administrations of IVIG or acetaminophen, only the first administration was considered. The dose of IVIG or acetaminophen was determined at the discretion of the physician.

### Data collection

The following data were collected manually from electronic medical records: age, sex, body weight, comorbidities, infection site, sequential organ failure assessment (SOFA) score at the administration of target drugs, organ support at the administration of target drugs (invasive mechanical ventilation, renal replacement therapy, vasopressors), dose of target drugs, and concomitant drug use. In previous studies, hypotension in critically ill patients occurred for approximately 2 h [4, 5]. Therefore, the observation period was set to 2 h from the start of IVIG or acetaminophen administration. The following data were investigated at the start of, as well as 15, 30, 45, 60, 75, 90, 105, and 120 min after, administration: systolic blood pressure (SBP), diastolic blood pressure (DBP), mean arterial pressure (MAP), heart rate (HR), starting or increasing the use of vasopressors, and a fluid bolus of crystalloids exceeding 500 mL or colloids of more than 250 mL.

### Study endpoints

The primary endpoint was the incidence of hypotension within 2 h after the administration of the target drug. To ensure valid comparison with previous studies, hypotension was defined as a decrease in the MAP of greater than or equal to 15% from baseline [5, 18]. Secondary endpoints were the incidence of hypotension or therapeutic intervention for hypotension (starting or increasing the use of vasopressors, fluid bolus of crystalloids more than 500 mL, or colloids of more than 250 mL) for the 2 h after the administration of the target drug, and in-hospital mortality.

### Statistical analysis

Patient characteristics were analyzed using descriptive statistics. Categorical variables were compared using the Chi-squared test or Fisher exact test. Continuous variables were compared using the nonparametric Wilcoxon signed-rank test, Mann–Whitney U test, or Friedman test.

We performed logistic regression analyses to identify variables significantly associated with hypotension, as measured by the estimated odds ratio (OR) and 95% confidence interval (CI). Variables yielding *p* values less than 0.20 in the univariate analyses or considered clinically relevant were entered in a forward stepwise logistic regression model. Non–log-linear continuous variables were dichotomized. Covariates were entered into the model with critical entry and removed at *p* values of 0.20 and 0.1, respectively. Multicollinearity and interactions were tested. The Hosmer–Lemeshow test was used to check the goodness of fit of the logistic regression. All tests were two-sided, and *p* values less than 0.05 were considered statistically significant.

Furthermore, we performed a propensity score analysis with a propensity score matching method to adjust for treatment selection bias (IVIG or acetaminophen). Possible confounders were selected for their potential association with the treatment based on clinical knowledge. Variables included in the model were age, sex, body weight, comorbidities (hypertension, diabetes, chronic heart failure), infection site (pneumonia, bacteremia), invasive mechanical ventilation, renal replacement therapy, vasopressors, SOFA score at the administration of target drugs, and MAP at baseline. Propensity matching was performed using the nearest neighbor matching without replacement, with IVIG prescriptions matched 1:1 to acetaminophen prescriptions. For the developed propensity score, a caliper width of 0.2 of the standard deviation of the logit of propensity score was used. After matching, potential associations between treatment (IVIG or acetaminophen) and endpoints were evaluated using the Chi-squared test or Fisher exact test, analyzed at a significance level of 0.05. Standardized differences were used to assess the balance of confounders between treatments, as described by Peter C Austin [19]. A standardized difference < 0.1 suggested negligible differences in the mean or prevalence of covariates between treatments.

Based on previous findings [5], the sample size was estimated as follows. Using a two-sided Chi-squared test with 0.05 type I error and 0.2 type II error, we estimated that 200 patients (100 patients in IVIG group and 100 patients in acetaminophen group) were required to detect a significant change in MAP between baseline and lowest MAP, assuming a decrease of 15% of MAP occurring in 25% of the IVIG group and 50% of the acetaminophen group.

All statistical analyses were performed using SPSS software, v.25 (IBM, Armonk, NY, USA).

## Results

**Fig. 1:**
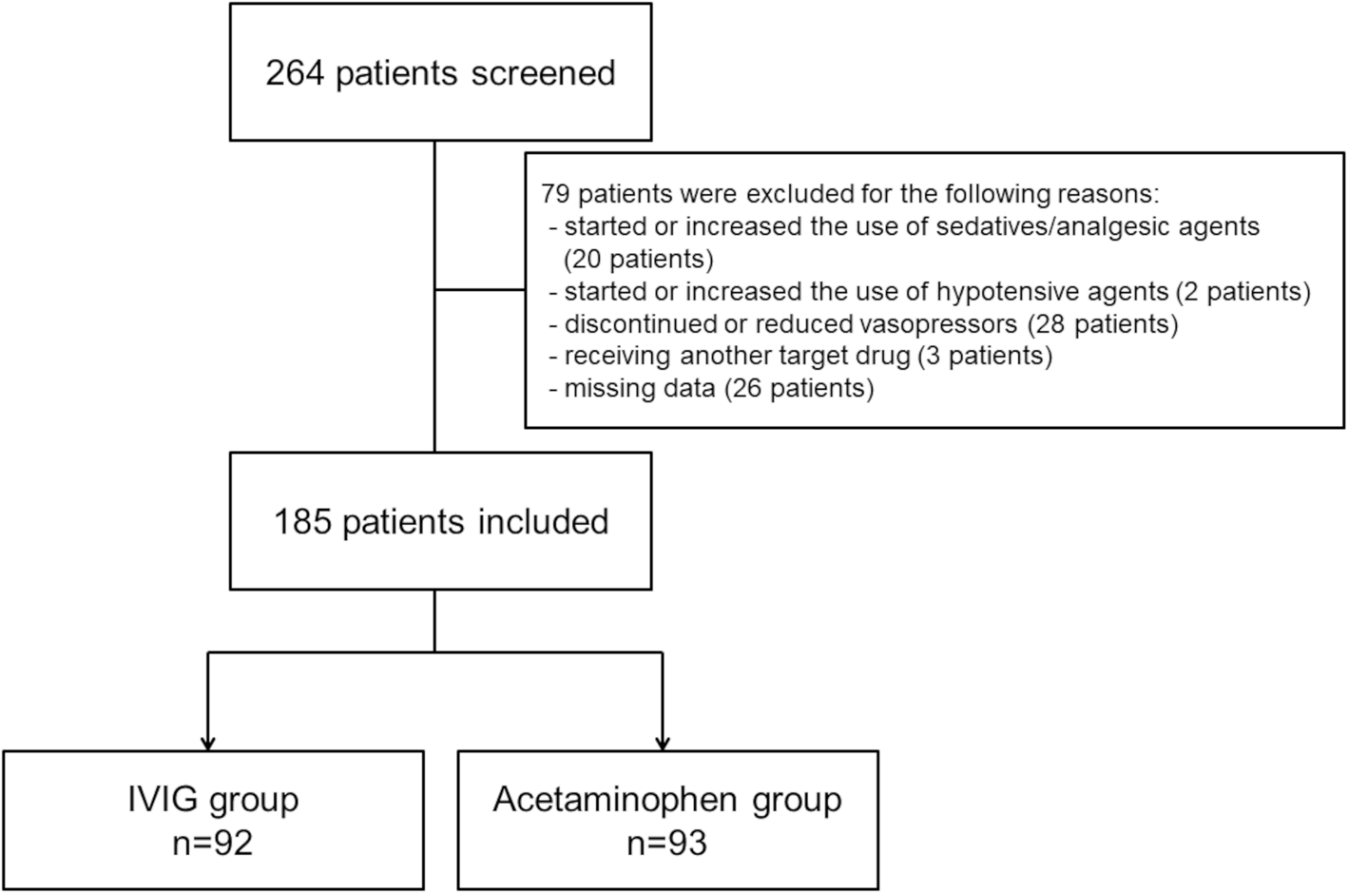
Patient selection process. *IVIG* intravenous immunoglobulin

Overall, 185 patients were included, with 92 and 93 patients in the IVIG and acetaminophen groups, respectively (Fig. 1). Baseline patient characteristics are listed in Table 1. The mean mannitol dose was 0.294 g/kg and 0.597 g/kg in the IVIG and acetaminophen groups, respectively. Patients in the acetaminophen group had lower SOFA scores than those in the IVIG group, and were less sick overall.

**Table 1:** Baseline characteristics of all patients

	IVIG (*n* = 92)		Acetaminophen (*n* = 93)		p
	n/mean	%/SD	n/mean	%/SD	
Female gender, n (%)	31	33.7%	23	24.7%	0.18
Age (yr)	70.1	14.7	64.2	17.7	0.01
Weight (kg)	56.0	11.2	61.2	12.4	< 0.01
Comorbidities, n (%)					
Hypertension	36	39.1%	35	37.6%	0.83
Diabetes	21	22.8%	17	18.3%	0.44
Chronic heart failure	7	7.6%	18	19.4%	0.02
Infection site, n (%)					
Pneumonia	33	35.9%	21	22.6%	0.05
Bacteremia	46	50.0%	30	32.3%	0.01
Intra-abdominal	26	28.3%	20	21.5%	0.29
SOFA score (median/quartile)	8	(11–14)	4	(6–8)	< 0.01
Organ support, n (%)					
Invasive mechanical ventilation	68	73.9%	55	59.1%	0.03
Renal replacement therapy	52	56.5%	28	30.1%	< 0.01
Vasopressors	65	70.7%	18	19.4%	< 0.01
Drugs, n (%)					
Diuretics	6	6.5%	18	19.4%	< 0.01
Nicardipine	5	5.4%	10	10.7%	0.19
Plasma creatinine (mg/dL)	1.95	1.44	1.64	1.82	0.21
Vital sign at IVIG/acetaminophen infusion					
SBP (mmHg)	109.0	27.0	128.8	28.2	< 0.01
MAP (mmHg)	74.6	18.4	82.6	17.0	< 0.01
DBP (mmHg)	55.6	15.1	59.6	14.1	0.06
HR (beats/min)	101.1	20.8	101.9	22.2	0.79

*IVIG* intravenous immunoglobulin, *SOFA* sequential organ failure assessment, *SBP* systolic blood pressure, *MAP* mean arterial pressure, *DBP* diastolic blood pressure, *HR* heart rate, *SD* standard deviation

**Fig. 2:**
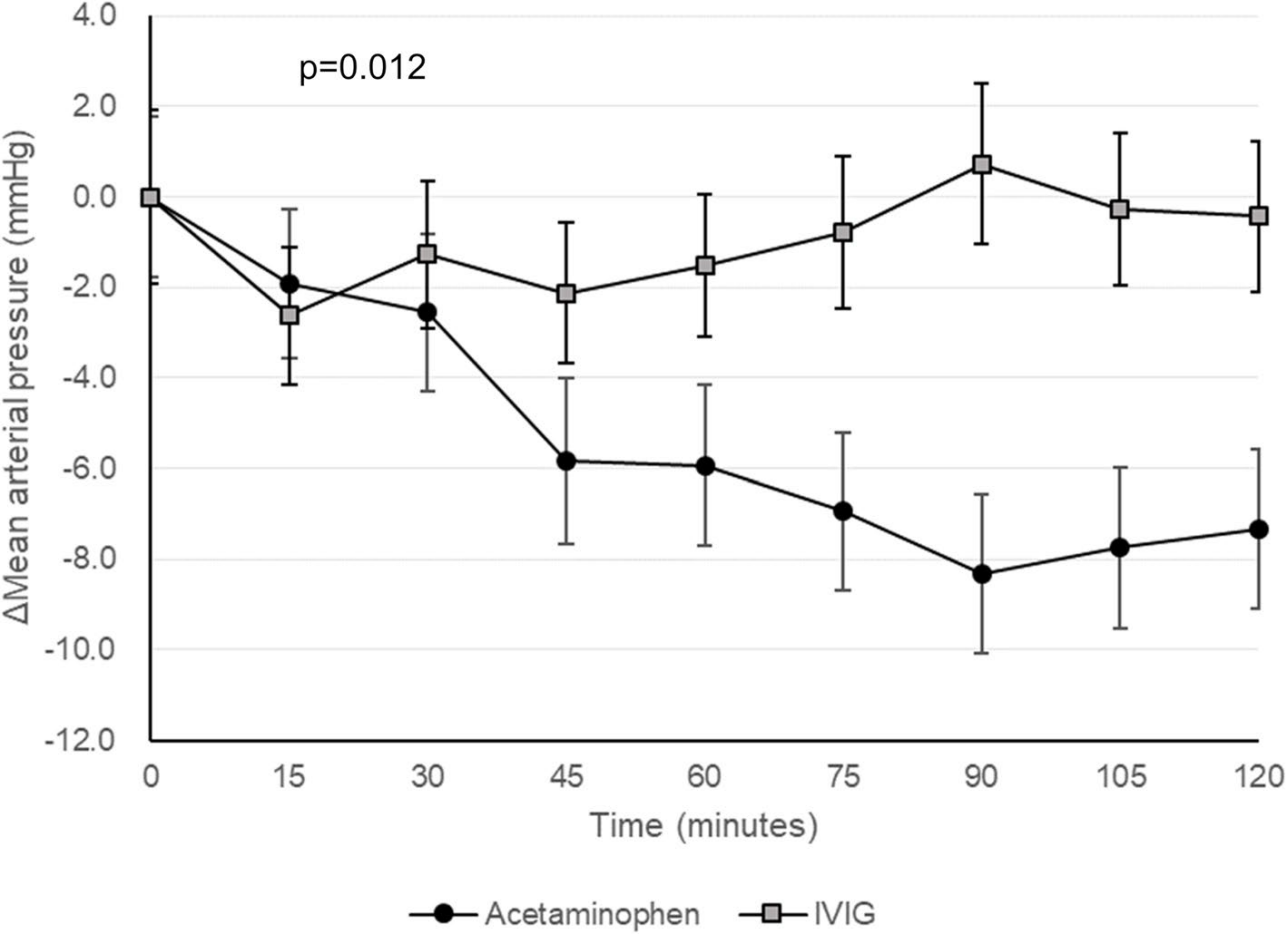
Changes in mean arterial pressure in the intravenous immunoglobulin (IVIG) and acetaminophen groups (mean ± standard error). Groups were compared using repeated analysis of variance

In the univariate analysis, the incidence of hypotension was higher in the acetaminophen group (58.0%; 54 of 93 patients) than in the IVIG group (36.9%; 34 of 92 patients) (OR = 8.26, *p* = 0.004). The incidence of hypotension or therapeutic intervention for hypotension was 45.6% in the IVIG group (42 of 92 patients) and 59.1% in the acetaminophen group (55 of 93 patients), but there was no significant difference (OR = 3.37, *p* = 0.066). The mean decrease in the MAP after administration is shown in Fig. 2. In the acetaminophen group, a statistically significant decrease in MAP was observed from 30 to 120 min. The mean change in the HR were -7.47 beats per minute in the IVIG group and -9.65 beats per minute in the acetaminophen group (*p* = 0.58).

In the acetaminophen group, the change in SBP (*r* = 0.738, *p* < 0.01) and DBP (*r* = 0.867, *p* < 0.01) demonstrated a significant correlation with MAP, whereas mannitol dose (*r* = 0.052, *p* = 0.70), acetaminophen dose (*r* = 0.052, *p* = 0.70), and SOFA score (*r* = 0.186, *p* = 0.17) had no significant correlation with MAP.

In multivariate logistic regression analyses to identify variables associated with hypotension, acetaminophen administration, high positive end-expiratory pressure of mechanical ventilation at baseline and high MAP at baseline were associated with hypotension (Table 2). In multivariate logistic regression analyses to identify variables associated with hypotension or therapeutic intervention for hypotension, acetaminophen administration and renal replacement therapy were associated with hypotension or therapeutic intervention for hypotension (Table 2).

**Table 2:** Multivariate logistic regression analyses to identify variables associated with hypotension and hypotension or therapeutic intervention for hypotension

	OR	95% CI	p
Variables associated with hypotension			
Acetaminophen administration	3.83	1.72–8.54	0.001
High positive end-expiratory pressure	2.01	1.03–3.90	0.039
High mean arterial pressure at baseline	1.89	1.01–3.56	0.048
Vasopressors	1.89	0.89–4.03	0.099
Pneumonia	0.56	0.28–1.12	0.099
Diuretics	0.41	0.15–1.09	0.073
Variables associated with hypotension or therapeutic intervention for hypotension			
Acetaminophen administration	4.03	1.80–8.98	0.001
Renal replacement therapy	2.37	1.22–4.58	0.011
Chronic heart failure	0.33	0.13–0.86	0.023
Vasopressors	2.03	0.96–4.28	0.064
Elder	1.70	0.90–3.20	0.102

*OR* odds ratio, *CI* confidence interval

In the propensity score-matched cohort, 80 matched patients were selected (Table 3). After matching, the confounders between treatment groups were well-balanced. The incidence of hypotension was 37.5% in the IVIG group (15 of 40 patients) and 67.5% in the acetaminophen group (27 of 40 patients) (OR = 7.21, *p* = 0.007). The incidence of hypotension or therapeutic intervention for hypotension was 40.0% in the IVIG group (16 of 40 patients) and 70.0% in the acetaminophen group (28 of 40 patients) (OR = 7.27, *p* = 0.007).

**Table 3:** Baseline characteristics of propensity score-matched patients

	IVIG (*n* = 40)		Acetaminophen (*n* = 40)		p
	n/mean	%/SD	n/mean	%/SD	
Female gender, n (%)	10	25.0%	8	20.0%	0.59
Age (yr)	66.7	15.6	67.1	16.4	0.91
Weight (kg)	56.0	10.5	59.8	10.4	0.11
Comorbidities, n (%)					
Hypertension	16	40.0%	18	45.0%	0.65
Diabetes	8	20.0%	8	20.0%	1.00
Chronic heart failure	6	15.0%	5	12.5%	0.75
Infection site, n (%)					
Pneumonia	11	27.5%	9	22.5%	0.61
Bacteremia	18	45.0%	18	45.0%	1.00
Intra-abdominal	13	32.5%	9	22.5%	0.32
SOFA score (median/quartile)	8	(6–11.25)	7	(5.75–9.25)	0.17
Organ support, n (%)					
Invasive mechanical ventilation	22	55.0%	24	60.0%	0.65
Renal replacement therapy	16	40.0%	17	42.5%	0.82
Vasopressors	17	42.5%	15	37.5%	0.65
Drugs, n (%)					
Diuretics	2	5.0%	6	15.0%	0.26
Nicardipine	5	12.5%	4	10.0%	0.72
Plasma creatinine (mg/dL)	1.97	1.57	1.85	1.98	0.77
Vital sign at IVIG/acetaminophen infusion					
SBP (mmHg)	114.4	30.4	119.4	22.4	0.41
MAP (mmHg)	76.5	19.8	77.2	16.2	0.86
DBP (mmHg)	56.7	16.6	56.1	13.6	0.84
HR (beats/min)	99.6	21.0	104.1	22.2	0.36

*IVIG* Intravenous immunoglobulin, *SOFA* Sequential organ failure assessment, *SBP* Systolic blood pressure, *MAP* Mean arterial pressure, *DBP* Diastolic blood pressure, *HR* Heart rate, *SD* Standard deviation

In multivariate logistic regression analyses to identify variables associated with hypotension after the administration of acetaminophen, high MAP at baseline was correlated with hypotension attributed to acetaminophen administration (OR = 2.70, 95%CI = 1.03–7.04). The acetaminophen dose, speed of acetaminophen infusion, and SOFA score at acetaminophen administration were not associated with hypotension after the administration of acetaminophen.

Overall, in-hospital mortality was higher in the IVIG group than in the acetaminophen group (41.3% vs 25.8%). However, in the propensity-matched cohort, a significant difference was not observed (27.5% vs 32.5%). Regarding in-hospital mortality, no difference was observed between patients who experienced a drop in blood pressure after acetaminophen administration and those who did not (20.4% vs 33.3%, *p* = 0.23).

## Discussion

In the present study, hypotension occurred in approximately 60% of patients with sepsis receiving acetaminophen treatment. In both univariate and multivariate analyses, acetaminophen administration considerably increased the incidence of hypotension compared with IVIG administration. The results were consistent with the analysis performed with propensity score matching to adjust the patient background and the severity of infection. In univariate analysis, the incidence of hypotension or therapeutic intervention for hypotension was not different between the groups, but the analysis was not sufficiently powered to detect statistically significant differences. Acetaminophen increased the incidence of hypotension more than IVIG, with both agents containing mannitol. Based on these results, it is postulated that acetaminophen formulation-induced hypotension is not attributed to the mannitol present, but rather to the action of acetaminophen itself.

The incidence of hypotension owing to acetaminophen administration varies widely [2–10], with several reasons for the diverse results reported, including small sample size, different definitions of hypotension, and differing patient severity. Aymeric Cantais et al. conducted a multicenter, prospective, observational study examining acetaminophen-induced hypotension in 160 severely ill patients, including 59 patients with sepsis. The incidence of hypotension in critically ill patients was approximately 50%, and it was reported that hypotension continued for about 2 h [5]. In the present study, we employed the definition of hypotension used by Cantais et al. to examine only patients with sepsis; the incidence of hypotension in the acetaminophen group was consistent with that in previous studies [5, 20]. Hyun Jong Lee et al. reported a high MAP at administration as a risk factor for hypotension following acetaminophen administration in relatively young patients with influenza A [10]. In the current study, high MAP at baseline was identified as a risk factor for hypotension following acetaminophen administration in sepsis.

The mechanism underlying the decreased blood pressure following acetaminophen administration remains elusive. It is speculated that the diuretic effect of mannitol, used as a stabilizing compound in acetaminophen formulations, can impact the decrease in blood pressure owing to acetaminophen administration in critically ill patients. In a randomized control trial comparing hemodynamic changes following the administration of acetaminophen, mannitol, and saline in healthy volunteers, Elizabeth Chiam et al. revealed that acetaminophen produces a higher rate of hypotension than mannitol; however, the study was small and did not include critically ill patients [4]. We conducted a retrospective study in critically ill patients with sepsis and observed that acetaminophen induced more hypotension than IVIG, both of which contained mannitol. Kelly et al., in a randomized controlled trial comparing the hemodynamic effects of parenteral and enteral acetaminophen in critically ill patients, showed that there is no difference in the incidence of hypotension between groups; however, the incidence of hypotension associated with acetaminophen administration is higher than that with parenteral acetaminophen [6]. In this study, blood pressures were observed only within 1 h after administration. We consider that blood concentrations may not have been sufficiently elevated in the enteral acetaminophen group. Therefore, the effect of mannitol in intravenous acetaminophen formulations could not be evaluated. Furthermore, in a small observational study using a transpulmonary thermodilution device by Adéla Krajčová et al., a decrease in systemic vascular resistance index (SVRI) reportedly observed after acetaminophen administration [20]. Assuming that the diuretic effect of mannitol causes a decrease in blood pressure, the decrease in blood pressure is probably attributed to hypovolemia owing to increased urine volume [21]; therefore, a decrease in SVRI should not be observed after acetaminophen administration. Reportedly, acetaminophen relieves cell-free hemoglobin-induced oxidative stress in patients with sepsis [22]. It has been suggested that cell-free hemoglobin may be involved in peripheral vasodilation via nitric oxide [23], which may be responsible for the decrease in blood pressure induced by acetaminophen. Another possible mechanism is that the decrease in sympathetic nerve stimulation owing to the relief of pain and discomfort may relax peripheral blood vessels [2, 6]. In recent years, basic studies in rats have revealed that N-acetyl-p-benzoquinone imine, an acetaminophen metabolite, directly causes vasodilation [24]. However, whether hypotension in humans occurs by a similar mechanism needs to be comprehensively investigated, and further research is warranted to elucidate the mechanism of acetaminophen-induced hypotension.

Our study is the largest to demonstrate changes in hemodynamics induced by acetaminophen in critically ill patients with sepsis, and its detection power is sufficient. To the best of our knowledge, there are no previous reports with sufficient power examining whether or not acetaminophen-induced hypotension in critically ill patients can be attributed to mannitol. We used propensity score matching to adjust patient background factors in treatment selection. Even in the matched cohort, the greater decrease in blood pressure in the acetaminophen group was unchanged, indicating the robustness of the result.

This study has some limitations. First, this was a retrospective chart review from a single center. The data was captured manually by authors who were not blinded. Therefore, the impact of several biases and confounding factors cannot be excluded. We performed both multivariate logistic regression analysis and propensity score matching, and the results were similar. However, it is undeniable that an unknown confounding factor may have contributed to hypotension. Patients did not receive acetaminophen randomly, but rather because of perceived clinical indications. For example, if the agent was administered for fever, the processes associated with fever, and not the drug, could be responsible for the hypotension. Second, as IVIG and acetaminophen differed in mannitol content, the mannitol dose differed between groups, and the balance could not be adjusted statistically, even using propensity score matching. Therefore, it is possible that the presence of a larger amount of mannitol in the acetaminophen group than in the IVIG group contributed to the decreased blood pressure. However, the mannitol dose and change in blood pressure failed to correlate with both the IVIG and acetaminophen groups. In multivariate logistic regression analyses, the mannitol dose was not associated with hypotension. Based on these results, it appears unlikely that the difference in the mannitol dose contributed to the decrease in blood pressure. Third, although IVIG provides mannitol like acetaminophen, it is a significant source of protein that is not present in the acetaminophen group, and the protein contained in IVIG may have affected hemodynamics. Moreover, it cannot be completely ruled out that the components contained in IVIG have an effect of suppressing the decrease in blood pressure and offset the negative effects of mannitol. Fourth, no difference in in-hospital mortality was observed between patients who experienced a drop in blood pressure and those who did not. We failed to demonstrate that acetaminophen-induced hypotension could contribute to patient outcomes, necessitating further studies to assess the deleterious effects of acetaminophen administration in critically ill patients.

## Conclusions

In our study, hypotension occurred in approximately 60% of patients with sepsis receiving acetaminophen treatment. In both univariate and multivariate analyses, acetaminophen administration enhanced the incidence of hypotension more than IVIG administration. The results were consistent with the analysis performed by propensity score matching with the adjusted patient background. Acetaminophen induced more hypotension than IVIG, with both agents containing mannitol. As IVIG and acetaminophen differed in mannitol content, the mannitol dose differed between groups. However, the mannitol dose and change in blood pressure failed to correlate with both the IVIG and acetaminophen groups. Further studies are needed to clarify the effects on hemodynamics of mannitol contained in acetaminophen formulations.

## Data Availability

The datasets used and/or analyzed during the current study are available from the corresponding author on reasonable request.

## Abbreviations

IVIG: Intravenous immunoglobulin
SOFA: Sequential organ failure assessment
SBP: Systolic blood pressure
DBP: Diastolic blood pressure
MAP: Mean arterial pressure
HR: Heart rate
OR: Odds ratio
CI: Confidence interval
SVRI: Systemic vascular resistance index
